# Dissociation of somatostatin and parvalbumin interneurons circuit dysfunctions underlying hippocampal theta and gamma oscillations impaired by amyloid β oligomers in vivo

**DOI:** 10.1007/s00429-020-02044-3

**Published:** 2020-02-27

**Authors:** Hyowon Chung, Kyerl Park, Hyun Jae Jang, Michael M. Kohl, Jeehyun Kwag

**Affiliations:** 1 Department of Brain and Cognitive Engineering, Korea University, Seoul, Korea; 2 Department of Physiology, Anatomy and Genetics, University of Oxford, Oxford, UK

**Keywords:** Hippocampus, Somatostatin interneuron, Parvalbumin interneuron, Amyloid beta oligomers, Network oscillations, Alzheimer's disease

## Abstract

Accumulation of amyloid β oligomers (AβO) in Alzheimer’s disease (AD) impairs hippocampal theta and gamma oscillations. These oscillations are important in memory functions and depend on distinct subtypes of hippocampal interneurons such as somatostatin-positive (SST) and parvalbumin-positive (PV) interneurons. Here, we investigated whether AβO causes dysfunctions in SST and PV interneurons by optogenetically manipulating them during theta and gamma oscillations in vivo in AβO-injected SST-Cre or PV-Cre mice. Hippocampal in vivo multi-electrode recordings revealed that optogenetic activation of channelrhodopsin-2 (ChR2)-expressing SST and PV interneurons in AβO-injected mice selectively restored AβO-induced reduction of the peak power of theta and gamma oscillations, respectively, and resynchronized CA1 pyramidal cell (PC) spikes. Moreover, SST and PV interneuron spike phases were resynchronized relative to theta and gamma oscillations, respectively. Whole-cell voltage-clamp recordings in CA1 PC in ex vivo hippocampal slices from AβO-injected mice revealed that optogenetic activation of SST and PV interneurons enhanced spontaneous inhibitory postsynaptic currents (IPSCs) selectively at theta and gamma frequencies, respectively. Furthermore, analyses of the stimulus–response curve, paired-pulse ratio, and short-term plasticity of SST and PV interneuron-evoked IPSCs ex vivo showed that AβO increased the initial GABA release probability to depress SST/PV interneuron’s inhibitory input to CA1 PC selectively at theta and gamma frequencies, respectively. Our results reveal frequency-specific and interneuron subtype-specific presynaptic dysfunctions of SST and PV interneurons’ input to CA1 PC as the synaptic mechanisms underlying AβO-induced impairments of hippocampal network oscillations and identify them as potential therapeutic targets for restoring hippocampal network oscillations in early AD.

**Electronic supplementary material:**

The online version of this article (10.1007/s00429-020-02044-3) contains supplementary material, which is available to authorized users.

## Introduction

Alzheimer’s disease (AD) is a neurodegenerative condition characterized by progressive memory loss and cognitive decline (LaFerla et al. 2007; Selkoe 2002; Hardy and Selkoe 2002). Abnormal accumulation of amyloid β oligomers (AβO) has been implicated in the pathogenesis of early stage AD (Hardy and Selkoe 2002; Lambert et al. 1998; Lesne et al. 2006; McLean et al. 1999; Walsh and Selkoe 2004), causing synaptic dysfunctions (Lacor et al. 2004; Shankar et al. 2007, 2008; Hsieh et al. 2006; Kamenetz et al. 2003) including impairments to synaptic plasticity (Lambert et al. 1998; Walsh and Selkoe 2004; Walsh et al. 2002) and ultimately neuronal death (Alberdi et al. 2010; De Felice et al. 2007; Decker et al. 2010). Similarly, transgenic mice that model AβO-related pathologies of AD (Wang et al. 2002; Iaccarino et al. 2016; Verret et al. 2012; Martinez-Losa et al. 2018; Tomiyama et al. 2010) show impairments to synaptic plasticity (Chapman et al. 1999; Rowan et al. 2003; Larson et al. 1999; Tomiyama et al. 2010) and memory functions (Brouillette et al. 2012; Kim et al. 2014; Tomiyama et al. 2010). Especially, hippocampal network oscillations, which are important for spatial information processing (O'Keefe and Recce 1993; Dragoi and Buzsaki 2006) and synaptic plasticity (Huerta and Lisman 1993; Bikbaev and Manahan-Vaughan 2008; Buzsaki 2002; Vertes 2005), are impaired in these animal models of AD (Driver et al. 2007; Iaccarino et al. 2016; Ittner et al. 2014; Mondragon-Rodriguez et al. 2018; Palop and Mucke 2016; Villette et al. 2010; Wang et al. 2002). Distinct subtypes of hippocampal interneurons are critically involved in hippocampal oscillogenesis (Huh et al. 2016; Mikulovic et al. 2018; Amilhon et al. 2015; Gulyas et al. 2010; Mann et al. 2005; Wang and Buzsaki 1996). Somatostatin-positive (SST) interneurons preferentially modulate theta oscillations (Mikulovic et al. 2018), while parvalbumin-positive (PV) interneurons modulate gamma oscillations (Iaccarino et al. 2016; Huh et al. 2016; Amilhon et al. 2015). In addition, SST interneurons such as oriens lacunosum-moleculare (O-LM) cells show enhanced responses to theta frequency inputs (Pike et al. 2000; Zemankovics et al. 2010; Whittington and Traub 2003), while PV interneurons such as basket cells show enhanced responses to gamma frequency inputs (Pike et al. 2000; Zemankovics et al. 2010), further indicating their cell type-specific involvement in theta and gamma oscillations. Recent experimental evidence revealed that SST and PV interneurons disintegrate structurally and functionally in mouse models of AD (Palop et al. 2007; Schmid et al. 2016; Chen et al. 2018; Verret et al. 2012; Martinez-Losa et al. 2018; Iaccarino et al. 2016), suggesting that dysfunctions of SST and PV interneurons may underlie impairments of theta and gamma oscillations observed in AD. Optogenetic activation (Iaccarino et al. 2016) or molecular manipulation (Martinez-Losa et al. 2018; Zhang et al. 2017) of PV interneurons could restore impaired hippocampal gamma oscillations in mouse models of AD. However, the relative contribution of SST and PV interneurons to the impairments of hippocampal theta and gamma oscillations observed in AD remains unknown.

To address this, we optogenetically manipulated the activity of SST or PV interneurons in an AβO-injected mouse model of AD. We found that AβO injections into the hippocampus reduced the peak powers of theta and gamma oscillations and desynchronized the spike phases of CA1 pyramidal cells (PCs) relative to both theta and gamma cycles. Optogenetic activation of ChR2-expressing SST and PV interneurons selectively restored the peak power of theta and gamma oscillations, respectively, to the levels observed in the control mice, and resynchronized CA1 PC spike phases. Further analyses revealed that spike phases of SST and PV interneurons in AβO-injected mice were also resynchronized selectively relative to theta and gamma oscillations, respectively. Ex vivo whole-cell voltage-clamp recordings in CA1 PC in acute hippocampal slices from AβO-injected mice revealed that sustained optogenetic activation of SST and PV interneurons selectively enhanced spontaneous inhibitory postsynaptic currents (sIPSCs) onto CA1 PC at theta and gamma frequencies, respectively. Investigating the AβO-induced changes at the synaptic level through analyzing the stimulus–response (*S*–*R*) curve, paired-pulse ratio, and short-term plasticity revealed that AβO increased the initial GABA release probability resulting in the depression of SST and PV interneuron-evoked inhibitory postsynaptic currents (IPSCs) selectively at 5 Hz and 40 Hz, respectively. Together, these results suggest that interneuron subtype-specific and frequency-specific presynaptic dysfunctions of SST and PV interneurons’ inhibitory inputs to CA1 PC may underpin the impairment of theta and gamma oscillations in AβO-injected mice in vivo.

## Materials and methods

### Animals

All experimental animals were obtained from Jackson Laboratory (SST-IRES-Cre knock-in mice: stock no. 013044, PV-Cre knock-in mice: stock no. 017320, Jackson Laboratory, Bar Harbor, ME, USA) (Taniguchi et al. 2011). Mice were housed in a temperature-controlled environment under a 12:12 h light–dark cycle with food and water provided ad libitum. All experimental procedures were approved by the Institutional Animal Care and Use Committee (IACUC) of Korea University (KUIACUC-2017-112 and KUIACUC-2019-0068).

### Soluble AβO preparation

Soluble AβO was prepared following the methods described in (Lambert et al. 1998) with slight modification in Aβ oligomerization (Park et al. 2020). Soluble Aβ_1–42_ (Bechem) monomerization was achieved by dissolving it in 1,1,1,3,3,3-hexafluoro-2-propanol (HFIP, Sigma Aldrich) at a final concentration of 1 mM. The HFIP-Aβ_1–42_ solution was incubated at room temperature (90 min) and HFIP was evaporated using a vacuum evaporator (SpeedVac, N-BIOTEK Inc.). The remaining monomerized Aβ_1–42_ film was dissolved in dimethyl sulfoxide (DMSO, Sigma Aldrich) to make 50 μM Aβ_1–42_ stock. Aβ_1–42_ stock was aliquoted and stored at − 20 °C. One day before AβO injection surgery, the Aβ_1–42_ stock was thawed and diluted to a final concentration of 10 μM in PBS. After dilution, Aβ_1–42_ solution was incubated for 18 h for Aβ oligomerization.

### Stereotaxic surgery

**Fig. 1:**
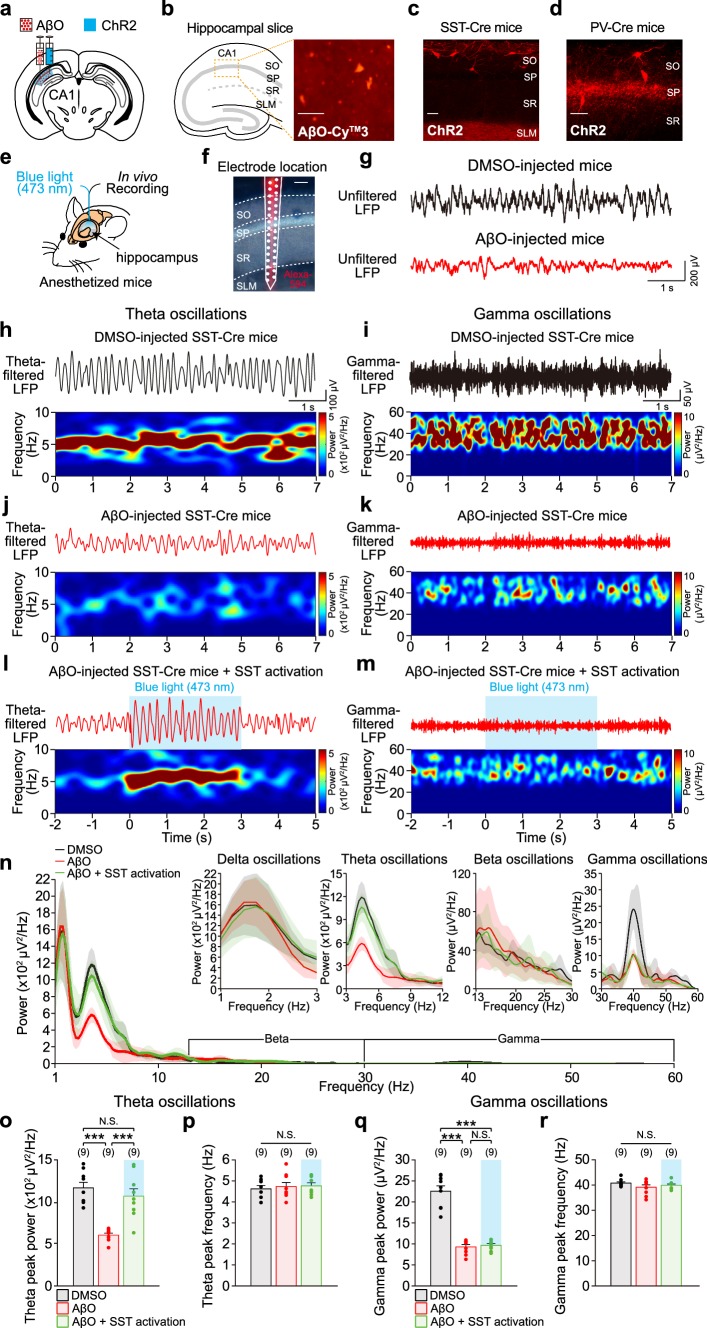
Optogenetic activation of SST interneurons selectively restores hippocampal theta oscillations impaired in AβO-injected mice in vivo. **a** Schematic illustration of AβO and ChR2-carrying AAV virus injection into hippocampal CA1 region unilaterally. **b** Left, schematic illustration of hippocampus structure. Right, immunochemistry showing deposition of AβO in sub-layers of CA1 (SO: stratum oriens, SP: stratum pyramidale, SR: stratum radiatum, SLM: stratum lacunosum-moleculare). Scale bar: 100 μm. Fluorescence image of ChR2-expressing SST interneurons in AβO-injected SST-Cre mice (**c**) and PV interneurons in AβO-injected PV-Cre mice (**d**). Scale bars: 100 μm. **e** Schematic illustration of in vivo recording and blue light (473 nm) stimulation in CA1 region of the hippocampus in anesthetized mice. **f** Position of the 32-channel silicon probe in CA1 region. Alexa 594 fluorescent dye-stained probe track identified the sub-layers of CA1. Signals from SP were used for LFP analysis. Scale bar 100 μm. **g** Representative traces of unfiltered LFPs recorded in DMSO-injected (top, black trace) and AβO-injected SST-Cre mice (bottom, red trace). Representative traces of band-pass filtered LFPs (top) at theta (**h**) and gamma frequency (**i**) and the corresponding power spectrograms (bottom) recorded from DMSO-injected SST-Cre mice. **j, k** Same as **h**, **i** but in AβO-injected SST-Cre mice. **l**, **m** Same as **h**, **i** but with blue light stimulation of ChR2-expressing SST interneuron in AβO-injected SST-Cre mice. **n** Power spectral density (PSD) of unfiltered LFPs (shade indicates SEM) recorded in DMSO-injected SST-Cre mice (*n* = 9, black), AβO-injected SST-Cre mice (*n* = 9, red), and with blue light stimulation of ChR2-expressing SST interneuron in AβO-injected SST-Cre mice (*n* = 9, green). Insets show PSD of unfiltered LFPs in delta, theta, beta, and gamma frequency-ranges. Mean peak power (**o**) and mean peak frequency (**p**) of theta oscillations analyzed from the PSD of each condition in **n**. **q**, **r** Same as **o**, **p** but for gamma oscillations in each condition. *n* The number of animals from which LFPs were recorded. One-way ANOVA followed by Tukey’s post hoc test (**o**–**r** ***p < 0.001, N.S. *p* > 0.05). Data are mean ± SEM with individual data values (dots)

To express channelrhodopsin-2 (ChR2) in SST or PV interneurons, we injected adeno-associated virus (AAV) (AAV5-EF1a-DIO-hChR2(E123T/T159C)-p2A-mCherry-WPRE, 3.8 × 10^12^ virus molecules/mL, 1 μL, UNC Vector Core) into the CA1 region of the hippocampus in SST-Cre and PV-Cre mice (postnatal day 28–49). AAV was injected unilaterally into the CA1 region of the left hippocampus (2.7 mm posterior, 2.7 mm lateral and 1.85 mm ventral from the bregma) using a 5 μL micro-needle (Hamilton Company) connected to a motorized stereotaxic injector (delivery rate: 0.1 μL/min). Then, either DMSO (diluted in PBS, 3 μL, delivery rate: 0.3 μL/min) or soluble AβO (3 μL, delivery rate: 0.3 μL/min) was co-injected to the same location to create control mice or mice with AβO pathology, respectively (Fig. 1a). To establish whether there is a difference between unilateral and bilateral hippocampal injections of AβO on hippocampal local field potentials (LFPs), we injected AβO in CA1 region of the hippocampus bilaterally as well (Online resource Fig. 1). The needle was left in the brain for > 5 min to minimize backflow of the injection. Body temperature was maintained using a heating pad during surgery. At least 3 weeks of recovery period was allowed after surgery before in vivo or ex vivo experiments to ensure the proper expression of the virus and induction of AβO pathology (Villette et al. 2010).

### Hippocampal in vivo recording

In vivo LFPs and single unit recordings were made in anesthetized mice [ketamine (75–100 mg/kg), medetomidine (1 mg/kg)], head-fixed into a stereotaxic frame, using a 32-channel silicon probe (A1x32-Poly2-5mm-50s-177, Neuronexus) inserted into the hippocampal CA1 region (2.7 mm posterior, 2.7 mm lateral from bregma, and 1.85 mm ventral from the pia) (Fig. 1e). Extracellular signals were sampled at 25 kHz for single unit acquisition and then down-sampled to 1 kHz for LFP acquisition (RZ2, Tucker-Davis Technologies). For the optogenetic activation of SST or PV interneurons, blue LED light stimulation (473 nm, 50% of maximum intensity, X-Cite 110LED, Excelitas Technologies) was delivered via an optical fiber laminated to the 32-channel silicon probe (3 s, inter-trial interval of 60 s, 10 repetitions) (Fig. 1e). Body temperature was monitored and maintained at 37 °C throughout the experiments.

### Immunohistochemistry

To determine the position of each electrode within the hippocampal subregions, the silicon probe was removed from the hippocampus after the in vivo recording and coated with a fluorescent dye (Alexa 594). Alexa 594-coated silicon probe was re-inserted to the same coordinates in the hippocampus at least for 1 min. Mice were then perfused with ice-cold cutting solution [(in mM): 180 sucrose, 2.5 KCl, 1.25 NaH_2_PO_4_, 25 NaHCO_3_, 11 glucose, 2 MgSO_4_, and 1 CaCl_2_ (pH 7.2–7.4, 280–290 mOsm, and oxygenated with 95% O_2_/5% CO_2_)], after which the removed mice brain was cut into 300 μm hippocampal slices in ice-cold cutting solution using a vibratome (VT 1000 S, Leica Microsystems) and fixed in 4% paraformaldehyde (PFA, Sigma-Aldrich) for > 24 h at 4 °C. Fixed slices were rinsed in wash buffer three times (0.3% Triton X-100 in 0.1 M PBS) and mounted on glass slides. Fluorescent signal of Alexa 594 was visualized using a fluorescent microscope (Leica DM2500, Fig. 1f). To verify the deposition of AβO in the hippocampal CA1 region, PFA-fixed hippocampal slices were incubated in peroxidase buffer (0.3% H_2_O_2_ in 0.1 M PBS) for 20 min. Non-targeted antigens were blocked by incubation in 6% bovine serum albumin and 0.3% Triton X-100 in 0.1 M PBS for > 24 h at 4 °C, after which slices were incubated with primary antibody (anti-beta amyloid 1–42 antibody [mOC64]-Conformation-Specific, Abcam, 1:300 dilution in 0.1 M PBS, at 4 °C for 4 days) and secondary antibody (Cy^TM^3 AffiniPure Donkey Anti-Rabbit IgG (H + L), Jackson ImmunoResearch, 1:500 dilution in 0.1 M PBS). Slices were washed and mounted on slide glasses with cubic mount (Lee et al. 2016) to clear the tissue. Immuno-stained AβO in the hippocampal slices were visualized with the fluorescent microscope (Leica DM2500, Fig. 1b). Expression of ChR2 in SST and PV interneurons was verified by detecting the fluorescent signal of the fused mCherry reporter using the fluorescent microscope (DM2500, Leica) or confocal microscope (LSM-700, ZEISS, Fig. 1c, d).

### Data analysis of in vivo LFPs and spikes

**Fig. 2:**
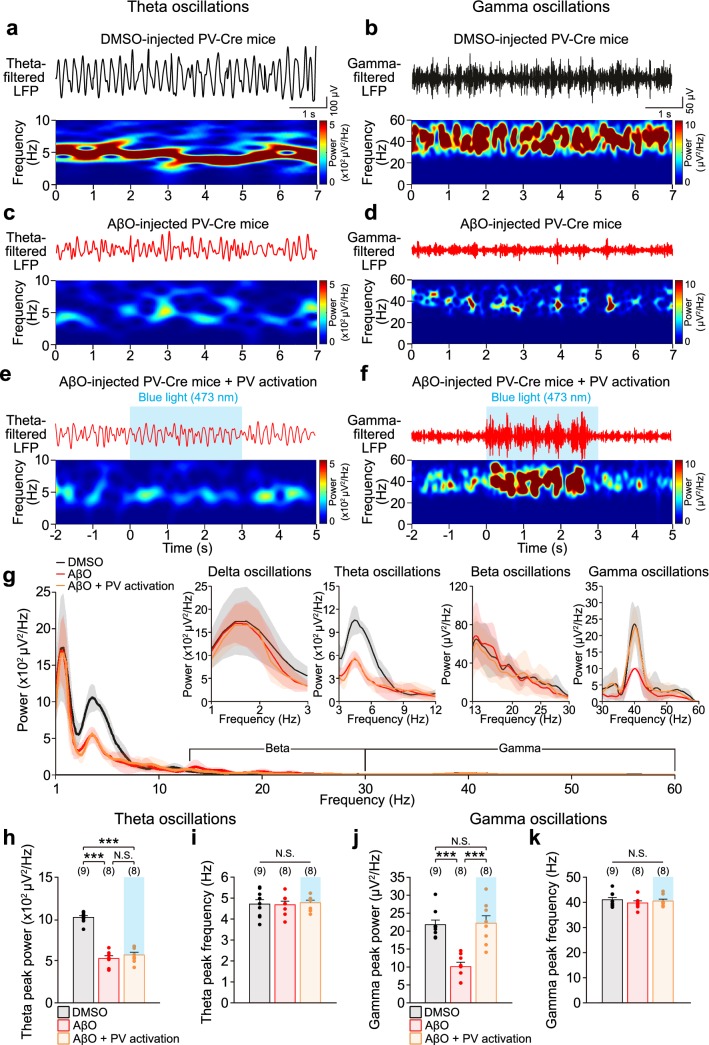
Optogenetic activation of PV interneurons restores hippocampal gamma oscillations impaired in AβO-injected mice in vivo. Representative traces of band-pass filtered LFPs (top) at theta (**a**) and gamma frequency (**b**) and corresponding power spectrograms (bottom) recorded from DMSO-injected PV-Cre mice. **c**,** d** Same as **a**, **b** but in AβO-injected PV-Cre mice. **e**, **f** Same as **a**, **b** but with blue light (473 nm) stimulation (blue shade) of ChR2-expressing PV interneurons in AβO-injected PV-Cre mice. **g** Power spectral density (PSD) of unfiltered LFPs (shade indicates SEM) recorded in DMSO-injected PV-Cre mice (*n* = 9, black), AβO-injected PV-Cre mice (*n* = 8, red), and with blue light stimulation of ChR2-expressing PV interneuron in AβO-injected PV-Cre mice (*n* = 8, orange). Insets show PSD of unfiltered LFPs in delta, theta, beta, and gamma frequency-ranges. Mean peak power (**h**) and mean peak frequency (**i**) of theta oscillations analyzed from the PSD of each condition in **g**. **j**, **k** Same as **h**, **i** but those of gamma oscillations in each condition. *n* The number of animals from which LFPs were recorded. One-way ANOVA followed by Tukey’s post hoc test (**h**–**k** ***p < 0.001, N.S. *p* > 0.05). Data are mean ± SEM with individual data values (dots)

**Fig. 3:**
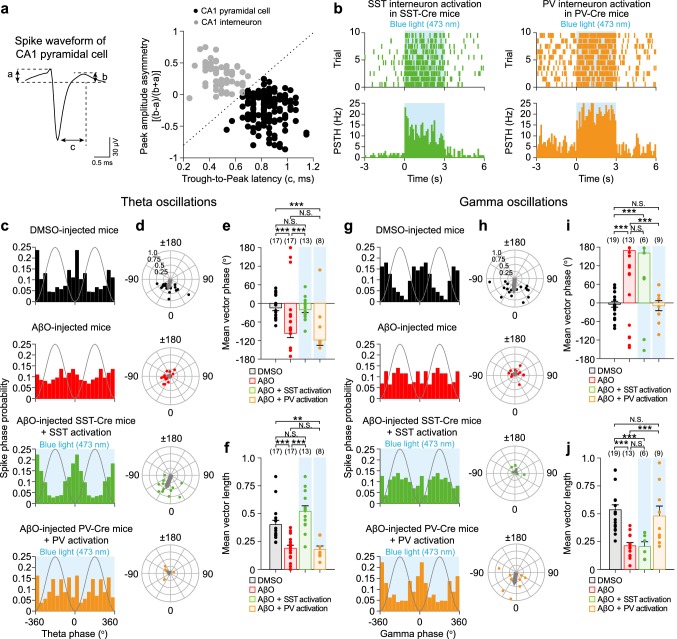
Optogenetic activation of SST and PV interneurons resynchronizes CA1 PC spike phases relative to theta and gamma oscillations, respectively, in AβO-injected mice in vivo. **a** Left, spike waveform analysis by calculating the early (*a*) and late part of (*b*) baseline-to-peak amplitude and trough-to-peak latency (*c*). Right, classification of single units into putative CA1 pyramidal cell (PC, black) and putative CA1 interneuron (gray) based on peak amplitude asymmetry [(*b* − *a*)/(*b* + *a*)] plotted as a function of *c*. Dashed line is the decision boundary for determining putative CA1 PC (right) and putative CA1 interneuron (left). **b** Spike raster plot (top) and peri-stimulus time histogram (PSTH, bottom) of ChR2-expressing SST (left, green) and ChR2-expressing PV interneuron (right, orange) in response to blue light (473 nm) stimulation (blue shade). The probability distribution of CA1 PC spike phases relative to theta oscillations (**c**) and polar plot of resultant vector phase/vector length (**d***,* filled circles) and the mean resultant vectors (**d**, gray arrow) analyzed from theta phase distribution of individual CA1 PC in DMSO-injected mice (*n* = 17, black), AβO-injected mice (*n* = 17, red), with blue light stimulation of ChR2-expressing SST interneuron in AβO-injected SST-Cre mice (*n* = 13, green), and with blue light stimulation of ChR2-expressing PV interneuron in AβO-injected PV-Cre mice (*n* = 8, orange). Mean phase (**e**) and mean length (**f**) of resultant vector. **g–j** Same as **c–f** but for CA1 PC spike phases relative to gamma oscillations recorded in DMSO-injected mice (*n* = 19, black), AβO-injected mice (*n* = 13, red), with blue light stimulation of ChR2-expressing SST interneuron in AβO-injected SST-Cre mice (*n* = 6, green), and with blue light stimulation of ChR2-expressing PV interneuron in AβO-injected PV-Cre mice (*n* = 9, orange). *n* The number of CA1 PC units. Watson-Williams multi-sample circular test (**e**, **i**, ***p < 0.001, N.S. *p* > 0.05) and one-way ANOVA followed by Tukey’s post hoc test (**f**, **j**, ***p < 0.001, **p < 0.01, N.S. *p* > 0.05). Data are mean ± SEM with individual data values (dots)

**Fig. 4:**
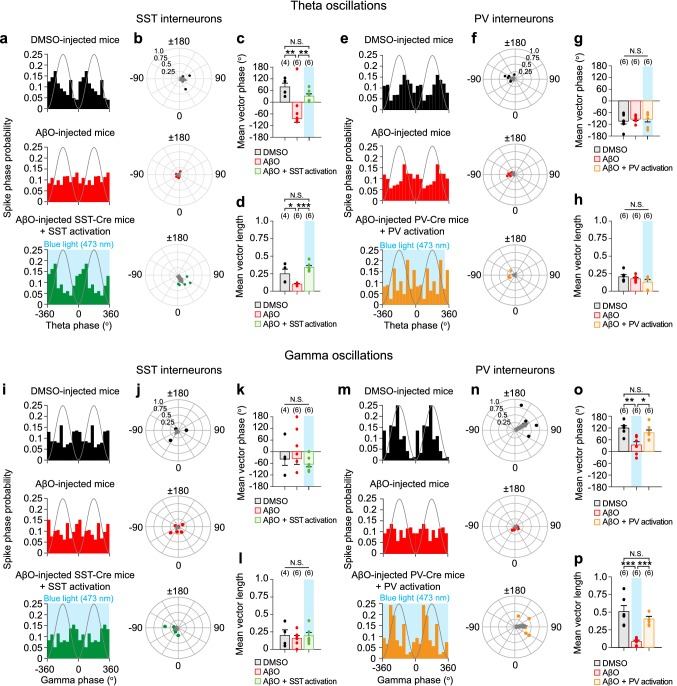
Optogenetic activation of SST and PV interneurons resynchronizes SST and PV interneuron spike phases relative to theta and gamma oscillations, respectively, in AβO-injected mice in vivo. The probability distribution of SST interneuron spike phases relative to theta oscillations (**a**) and polar plot of resultant vector phase/vector length (**b**, filled circles) and the mean resultant vectors (**b***,* gray arrow) analyzed from theta phase distribution of individual SST interneuron in DMSO-injected SST-Cre mice (*n* = 4, black), AβO-injected SST-Cre mice (*n* = 6, red), and with blue light (473 nm) stimulation (blue shade) of ChR2-expressing SST interneuron in AβO-injected SST-Cre mice (*n* = 6, green). Mean phase (**c**) and mean length (**d**) of resultant vector. **e–h** Same as in **a–d** but for PV interneuron spike phases relative to theta oscillations in DMSO-injected PV-Cre mice (*n* = 6, black), AβO-injected PV-Cre mice (*n* = 6, red), and with blue light stimulation of ChR2-expressing PV interneuron in AβO-injected PV-Cre mice (*n* = 6, orange). The probability distribution of SST interneuron spike phases relative to gamma oscillations (**i**) and polar plot of resultant vector phase/vector length (**j**, filled circles) and the mean resultant vectors (**j***,* gray arrow) analyzed from gamma phase distribution of individual SST interneuron in DMSO-injected SST-Cre mice (*n* = 4, black), AβO-injected SST-Cre mice (*n* = 6, red), and with blue light stimulation of ChR2-expressing SST interneuron in AβO-injected SST-Cre mice (*n* = 6, green). Mean phase (**k**) and mean length (**l**) of resultant vector. **m–p** Same as in **i–l** but for PV interneuron spike phases relative to gamma oscillations in DMSO-injected PV-Cre mice (*n* = 6, black), AβO-injected PV-Cre mice (*n* = 6, red), and with blue light stimulation of ChR2-expressing PV interneuron in AβO-injected PV-Cre mice (*n* = 6, orange). *n* The number of SST or PV interneuron units. Watson-Williams multi-sample circular test (**c**, **g**, **k**, **o**, **p < 0.01, *p < 0.05, N.S. *p* > 0.05) and one-way ANOVA followed by Tukey’s post hoc test (**d**, **h**, **l**, **p**, ***p < 0.001, *p < 0.05, N.S. *p* > 0.05). Data are mean ± SEM with individual data values (dots)

LFP signals were common-average filtered at 1–500 Hz, after which a fast Fourier transform (FFT) was performed to analyze the power spectral density (PSD) of unfiltered LFPs (Figs. 1n, 2g; Online resource Figs. 1, 3, 4). Peak frequencies and peak powers of delta (1–3 Hz), theta (3–12 Hz), beta (13–30 Hz), and gamma oscillations (30–60 Hz) were characterized from the PSD of unfiltered LFPs at each frequency band (Figs. 1o–r, 2h–k; Online resource Figs. 1–4). Power spectrograms of theta and gamma oscillations were plotted by taking short-time Fourier transform to the third order butterworth bandpass filters (3–12 Hz for theta and 30–60 Hz for gamma oscillations, Figs. 1h–m, 2a–f; Online resource Figs. 1, 3, 4). Spikes were extracted from 300 to 5000 Hz band-pass filtered extracellular signals using the Klusta-suite software (Rossant et al. 2016). Spike waveform, inter-spike intervals, and the shape of auto-correlogram of spike times were examined to refine single unit identification (Hill et al. 2011). Classification of putative CA1 PCs and putative CA1 interneurons were based on spike waveform asymmetry index {[(*b* − *a*)/(*b* + *a*)], baseline-to-peak amplitude (*a*), last baseline-to-peak amplitude (*b*)} to the trough-to-peak latency (*c*). Neurons located on the right of the decision boundary ([(*b* − *a*)/(*b* + *a*)] = 2 × *c* − 1.2) were considered putative CA1 PCs (Fig. 3a). Putative CA1 interneurons that showed increased firing rates in response to blue light stimulation in SST-Cre or PV-Cre mice in the peri-stimulus time histogram (PSTH, 100 ms bin) were identified as putative SST or PV interneurons, respectively (Fig. 3b). Oscillation cycles were analyzed using the Hilbert transform and the spike times of each neuronal types were used to calculate the spike phases relative to theta or gamma cycles (Khodagholy et al. 2017; Tort et al. 2010). The probability distribution of spike phases was analyzed through normalizing the spike phases in each 36-degree bin by the total number of spikes (Figs. 3c, g, 4a, e, i, m). Each spike phase probability was vectorized on the polar coordinate (Figs. 3d, h, 4b, f, j, n). The mean spike phases were analyzed using the Circular Statistics Toolbox in MATLAB (Berens 2009) (Figs. 3e, i, 4c, g, k, o). The strength of phase-locking was analyzed by calculating the mean vector length of the spike phases on the polar coordinate (Figs. 3f, j, 4d, h, l, p) (Zar 1999). All LFP signals were analyzed using customized protocols in MATLAB (R2018a).

### Brain slice preparation for ex vivo recording

After 3 weeks following co-injection of AβO and ChR2-carrying AAV, mice were anesthetized with 1.25% avertin solution (8 g of 2,2,2-tribromoethanol, 5.1 mL of 2-methyl-2-butanol and 402.9 mL saline, Sigma Aldrich) and perfused with ice-cold cutting solution, after which the brain was rapidly removed into oxygenated ice-cold cutting solution. Coronal hippocampal slices (300 μm) were cut using a vibratome and allowed to recover for 20 min in a solution which was made up of a mixture of cutting solution and artificial cerebro spinal fluid (aCSF) [(in mM): 126 NaCl, 3 KCl, 1.25 NaH_2_PO_4_, 2 MgSO_4_, 2 CaCl_2_, 25 NaHCO_3_, and 10 glucose (pH 7.2–7.4, 280–290 mOsm, and oxygenated with 95% O_2_/5% CO_2_)] at 1:1 ratio. Slices were further incubated in oxygenated aCSF solution for at least 1 h at 30–32 °C before being transferred to the recording chamber.

### Ex vivo whole-cell patch-clamp recordings

**Fig. 5:**
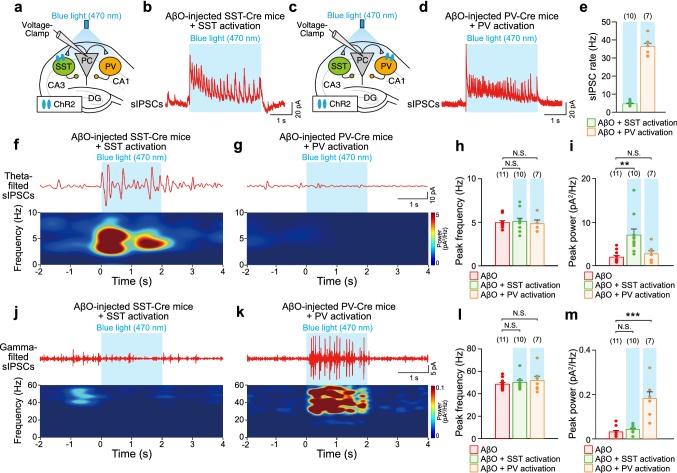
Sustained optogenetic activation of SST and PV interneurons in AβO-injected mice activates sIPSCs selectively at theta and gamma frequencies. **a** Schematic illustrating whole-cell voltage-clamp recordings of sIPSCs from CA1 PC in ex vivo hippocampal slices from AβO-injected SST-Cre mice during sustained blue light (470 nm) stimulation of ChR2-expressing SST interneurons. **b** Representative trace of sIPSCs recorded in CA1 PC during blue light stimulation (blue shade) of ChR2-expressing SST interneurons. **c**, **d** Same as **a**, **b** but sIPSCs recorded in CA1 PC during blue light stimulation of ChR2-expressing PV interneurons in AβO-injected PV-Cre mice. **e** Mean rates of sIPSCs recorded during blue light stimulation of ChR2-expressing SST interneurons in AβO-injected SST-Cre mice (*n* = 10, green) and during blue light stimulation of ChR2-expressing PV interneurons in AβO-injected PV-Cre mice (*n* = 7, orange). Representative traces of band-pass filtered sIPSCs at theta frequency (top) and the corresponding power spectrograms (bottom) during sustained blue light stimulation of ChR2-expressing SST interneurons (**f**) and ChR2-expressing PV interneurons (**g**) in ex vivo hippocampal slices from AβO-injected SST-Cre and PV-cre mice, respectively. Mean peak frequency (**h**) and mean peak power (**i**) of band-pass filtered sIPSCs at theta frequency during blue light stimulation of ChR2-expressing SST interneurons in AβO-injected SST-Cre mice mice (*n* = 10, green) and during blue light stimulation of ChR2-expressing PV interneurons in AβO-injected PV-Cre mice mice (*n* = 7, orange). **j–m** Same as **f–i** but for band-pass filtered sIPSCs at gamma frequency. *n* The number of cells from which sIPSCs were recorded. One-way ANOVA followed by Tukey’s post hoc test (**h**, **i**, **l**, **m**, ***p < 0.001, **p < 0.01, N.S. *p* > 0.05). Data are mean ± SEM with individual data values (dots)

**Fig. 6:**
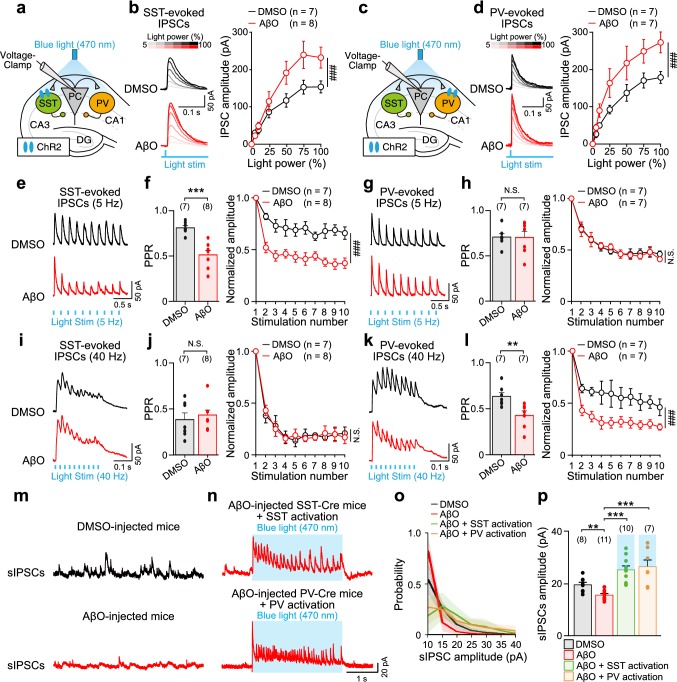
AβO induces presynaptic dysfunctions of SST and PV interneurons’ input to CA1 PC. **a** Schematic illustrating whole-cell voltage-clamp recordings of evoked-IPSCs from CA1 PC in response to blue light (470 nm) stimulation (blue shade) of ChR2-expressing SST interneurons in ex vivo hippocampal slices from DMSO-injected and AβO-injected SST-Cre mice. **b** Representative traces of SST interneuron-evoked IPSCs (SST-evoked IPSCs, left) recorded in CA1 PC and stimulus–response (*S*–*R*) curve (right) in response to different light stimulation intensities (5, 10, 25, 50, 75, and 100% of maximum power (15mW)) in hippocampal slices from DMSO-injected mice (*n* = 7, black), AβO-injected mice (*n* = 8, red). **c**, **d** Same as **a**,** b** but for PV interneuron-evoked IPSCs (PV-evoked IPSCs) recorded in CA1 PC (DMSO: *n* = 7, AβO: *n* = 7). Representative traces of SST-evoked IPSCs (**e**), paired-pulse ratio (PPR) (**f**, left), and SST-evoked-IPSCs normalized to the first evoked-IPSC showing short-term depression (**f**, right) in response to a train of ten pulses of blue light stimulation at 5 Hz in ex vivo hippocampal slices from DMSO-injected mice (*n* = 7, black) and AβO-injected mice (*n* = 8, red). **g**,** h** Same as **e–f** but with PV-evoked IPSCs recorded in CA1 PC (DMSO: *n* = 7, AβO: *n* = 7). **i–l** Same as **e**–**h** but in response to a train of ten pulses of blue light stimulation at 40 Hz. Representative traces of sIPSCs recorded in CA1 PC in the hippocampal slices from DMSO-injected mice (**m**, top, black), AβO-injected mice (**m**, bottom, red), AβO-injected mice during sustained blue light stimulation of ChR2-expressing SST interneurons (**n**, top, red), and ChR2-expressing PV interneurons (**n**, bottom, red). Probability distribution of sIPSC amplitudes (**o**) and mean amplitudes of sIPSCs (**p)** in each condition (DMSO: *n* = 8, AβO: *n* = 11, AβO + SST activation: *n* = 10, AβO + PV activation: *n* = 7). *n* The number of cells from which evoked IPSCs or sIPSCs were recorded. Two-way ANOVA followed by Tukey’s post hoc test (**b**, **d**, **f**, right, **h**, right, **j**, right, **l**, right, ^###^*p* < 0.001, N.S. *p* > 0.05), unpaired Student’s *t* test (**f**, left, **h**, left, **j**, left, **l**, left, ***p < 0.001, **p < 0.01, N.S. *p* > 0.05), and one-way ANOVA followed by Tukey’s post hoc test (**p**, ***p < 0.001, **p < 0.01). Data are mean ± SEM with individual data values (dots)

For ex vivo experiments, slices were moved to a recording chamber filled with aCSF at 30–32 °C. Whole-cell voltage-clamp recordings were performed on visually identified CA1 PC (BW51W, Olympus) using a borosilicate glass electrode (4–8 MΩ) filled with intracellular solution [(in mM): 115 cesium methanesulfonate, 8 NaCl, 10 HEPES, 0.3 Na_3_-GTP, 4 Mg-ATP, 0.3 EGTA, 5 QX-314, and 10 BAPTA (pH 7.3–7.4 and 280–290 mOsm)]. 10 min was allowed after break-through for stabilization before recordings commenced. Series resistance was monitored throughout the experiment and cells with > 20% change in series resistance over the course of the recording were discarded. IPSCs were recorded through voltage-clamp recordings from CA1 PC at a holding potential of + 10 mV. To record sIPSCs during sustained optogenetic activation of SST or PV interneurons, 3 s-long sustained blue light stimulation (470 nm, inter-trial interval of 60 s, 15 mW) was delivered (Figs. 5, 6; Online resource Fig. 5) using a digital micromirror device (DMD, Polygon400, Mightex). Detection threshold of sIPSCs was set at 10 pA, which was three-fold greater than the baseline noise standard deviation. Detection and analysis of sIPSCs were performed using customized MATLAB code. For power spectral analysis, sIPSCs were first down-sampled to 1 kHz before a band-pass filter was applied (3–12 Hz for theta frequency and 30–60 Hz for gamma frequency). Then, FFT was performed to analyze the PSD, from which the peak frequencies and the peak powers were characterized (Fig. 5h, i, l, m; Online resource Fig. 5f, g, j, k). Spectrograms of band-pass filtered sIPSCs at both theta and gamma frequencies were generated using a short-time Fourier transform. To avoid light stimulation-induced artifacts, the first 1 s of sIPSCs during sustained optogenetic activation of SST or PV interneurons was not included in the power analysis. To analyze the *S*–*R* curve of SST interneuron- and PV interneuron-evoked IPSCs (Fig. 6b, d), a single blue light pulse (470 nm, 5 ms) with different stimulation intensities [5, 10, 25, 50, 75, and 100% of maximal light power (15 mW)] was delivered to either ChR2-expressing SST or PV interneurons and the corresponding evoked IPSCs were recorded from CA1 PC. For the subsequent paired-pulse ratio (PPR) and short-term plasticity analysis (Fig. 6e–l), a train of ten blue light pulses (470 nm, 5 ms) with stimulation intensity that gave the half-maximal IPSC response in the *S*–*R* curve (3–9 mW) was delivered at either 5 or 40 Hz using DMD. PPR was calculated by normalizing the second evoked-IPSC by the first evoked-IPSC in the train (Fig. 6f, h, j, l, left). Short-term plasticity was analyzed by normalizing the evoked-IPSCs in the train to the first evoked-IPSC (Fig. 6f, h, j, l, right). All signals were amplified (MultiClamp 700B amplifier, Molecular Devices), low-pass filtered at 10 kHz, and acquired at 5 kHz using the ITC-18 data acquisition interface (HEKA Elektronik). Igor Pro software (WaveMetrics) was used for generating command signals, acquiring data as well as data analysis.

### Statistical analysis

Data are represented as mean ± standard error of the mean (SEM). Statistical tests include one-way or two-way ANOVA followed by Tukey’s post hoc test and Student’s *t* test. Statistical tests for spike phases were performed using Watson-Williams multi-sample circular test (Zar 1989). Statistical analyses were performed with either SPSS or MATLAB.

## Results

### Optogenetic activation of SST interneurons selectively restores hippocampal theta oscillations impaired in AβO-injected mice

To investigate the relative contribution of SST and PV interneurons to the impairment of hippocampal theta and gamma oscillations in AD, we created an AD mouse model by injecting AβO (Villette et al. 2010; Stephan et al. 2003, 2001; Walsh et al. 2002; Meyer-Luehmann et al. 2006) and co-injecting an AAV virus containing a Cre-dependent ChR2 (AAV5-Ef1a-DIO-hChR2(ET/TC)-mCherry) into hippocampal CA1 region unilaterally (Fig. 1a) as well as bilaterally (Online resource Fig. 1) in either SST-Cre or PV-Cre mice. This enabled us to selectively modulate ChR2-expressing SST or PV interneurons in mice with AβO pathology in vivo. Three weeks after injection, AβO deposits were detectable in the CA1 region (Fig. 1b) and ChR2 was expressed in SST and PV interneurons in AβO-injected SST-Cre (Fig. 1c) and PV-Cre mice (Fig. 1d). In these mice expressing AβO and ChR2s, we performed LFP recordings of the hippocampus in vivo using a silicon probe laminated with an optical fiber for delivering blue light (473 nm) to the recording site (Fig. 1e). LFP recordings were analyzed for neurons recorded in the stratum pyramidale (SP) layer of the hippocampus (Fig. 1f).

First, we investigated the contribution of SST interneurons to AβO-induced impairment of hippocampal theta and gamma oscillations in SST-Cre mice. The raw LFPs recorded from DMSO-injected (Fig. 1g, top) and AβO-injected SST-Cre mice (Fig. 1g, bottom) were band-pass filtered at theta (3–12 Hz) and gamma frequencies (30–60 Hz), after which the corresponding spectrograms were plotted, which revealed that the powers of both theta and gamma oscillations were reduced in AβO-injected SST-Cre mice compared to those in DMSO-injected SST-Cre mice (Fig. 1h–k). Similar reductions in the powers of theta and gamma oscillations were observed in mice that were injected with AβO in the hippocampus bilaterally (Online resource Fig. 1). In contrast, we found that AβO had no effect on the powers of delta (1–3 Hz) and beta oscillations (13–30 Hz; Online resource Fig. 2), indicating that the AβO-induced impairments were specific to theta and gamma oscillations. After confirming that the AβO-injected mice model can reliably replicate hippocampal theta and gamma oscillation impairments as observed in other studies AD (Driver et al. 2007; Iaccarino et al. 2016; Ittner et al. 2014; Mondragon-Rodriguez et al. 2018; Palop and Mucke 2016; Villette et al. 2010; Wang et al. 2002), we next investigated the effect of optogenetic activation of SST interneurons on theta and gamma oscillations in AβO-injected SST-Cre mice. Interestingly, 3-s long sustained blue light stimulation of ChR2-expressing SST interneurons in AβO-injected SST-Cre mice was able to restore the power of theta oscillations, while it had no effect on the power of impaired gamma oscillations, as shown in the band-pass filtered LFPs and the corresponding spectrograms (Fig. 1l, m). Power spectral density (PSD) of unfiltered LFPs (Fig. 1n) confirmed that the effect of optogenetic activation of SST interneurons was indeed specific to theta oscillations, while delta, beta, and gamma oscillations remained unaffected. To further quantify the effect of SST interneuron activation on theta and gamma oscillations, we analyzed the peak powers and peak frequencies of theta and gamma oscillations from the PSD. We found that the peak power of theta oscillations that was significantly reduced in AβO-injected SST-Cre mice was fully restored by the optogenetic activation of SST interneurons (Fig. 1o) without affecting the peak frequency of theta oscillations (Fig. 1p), while there was no effect on peak powers nor peak frequencies of delta, beta, and gamma oscillations (Fig. 1q, r; Online resource Fig. 2). These results indicate that SST interneurons selectively restored the power of theta oscillations impaired in AβO-injected mice.

### Optogenetic activation of PV interneurons selectively restores the hippocampal gamma oscillations impaired in AβO-injected mice

Next, we investigated the contribution of PV interneurons to the AβO-induced impairment of hippocampal theta and gamma oscillations in PV-Cre mice. Band-pass filtered LFPs and the corresponding spectrograms showed that the powers of both theta and gamma oscillations were also significantly reduced in AβO-injected PV-Cre mice compared to those in DMSO-injected PV-Cre mice (Fig. 2a–d). 3-s long sustained blue light stimulation of ChR2-expressing PV interneurons in AβO-injected PV-Cre mice had no effect on the impaired theta oscillations, while the power of gamma oscillations was restored as shown in the band-pass filtered LFPs and the corresponding spectrograms (Fig. 2e, f). The PSD of unfiltered LFPs (Fig. 2g) revealed that the effect of optogenetic activation of PV interneurons was specific to gamma oscillations as opposed to delta, theta and beta oscillations, which were all unaffected. The peak power of gamma oscillations that was significantly reduced in AβO-injected mice was fully restored by optogenetic activation of PV interneurons without affecting the peak frequency of gamma oscillations, while there was no effect on the peak powers and frequencies of delta, theta, and beta oscillations (Fig. 2h–k; Online resource Fig. 2).

Together, these results demonstrate that the AβO-induced decrease in the peak power of hippocampal theta and gamma oscillations can be selectively restored to control levels by optogenetic activation of SST and PV interneurons, respectively. In DMSO-injected control SST-Cre and PV-Cre mice, optogenetic activation of SST interneurons (Online resource Fig. 3) and PV interneurons (Online resource Fig. 4) also enhanced the peak power of theta and gamma oscillations, respectively, further supporting that distinct subtypes of interneurons are selectively involved in theta and gamma oscillations in both AD and the healthy brain.

### Optogenetic activation of SST and PV interneurons resynchronizes CA1 PC spike phases relative to theta and gamma oscillations, respectively, in AβO-injected mice

CA1 PC spikes preferentially occur at specific phases of hippocampal network oscillations and this synchronization is thought to be critical for spatial navigation and memory functions (Dragoi and Buzsaki 2006; Maurer and McNaughton 2007; O'Keefe and Recce 1993; Skaggs et al. 1996). Therefore, we next investigated the effect of AβO on CA1 PC spike synchrony with or without blue light stimulation of SST and PV interneurons. Recorded single units were divided into putative CA1 PCs and interneurons based on the spike waveforms (Fig. 3a), spike inter-spike intervals and autocorrelogram (see “Materials and methods”). Putative interneurons were further subdivided into putative SST and putative PV interneurons based on their increased firing rates in response to blue light stimulation in the peri-stimulus time histogram (PSTH, Fig. 3b). To quantify the strength of synchronization of CA1 PC spikes during theta oscillations, we analyzed the probability distribution of CA1 PC spike phases relative to the theta cycle in DMSO-injected mice and before/during blue light stimulation of SST and PV interneurons in AβO-injected mice. In DMSO-injected mice, CA1 PCs spike phases were strongly locked before the trough of the theta cycle (Fig. 3c–f, black), consistent with previous in vivo recordings in anesthesia states (Klausberger et al. 2003; Somogyi et al. 2014; Klausberger and Somogyi 2008). Theta spike phases of CA1 PC in AβO-injected mice occurred at a significantly earlier phase before the trough of theta oscillations (Fig. 3c–e, red) with reduced mean vector length of spike phase compared to those in DMSO-injected mice (Fig. 3c, d, f, red). However, blue light stimulation of SST interneurons in AβO-injected SST-Cre mice (Fig. 3c–f, green), but not PV interneuron in AβO-injected PV-Cre mice (Fig. 3c–f, orange), restored the mean spike phase (Fig. 3e) and the mean vector length of spike phase similar to those in the DMSO-injected mice (Fig. 3f).

The same CA1 PC spike phase analyses were repeated relative to gamma oscillations. In DMSO-injected mice, CA1 PCs spiked preferentially at the trough of the gamma cycle (Fig. 3g–j, black), consistent with previous in vivo recordings in anesthesia states (Tukker et al. 2007, 2013; Lasztoczi et al. 2011). In AβO-injected mice, gamma spike phases of CA1 PCs occurred at a significantly later phase after the trough of the gamma oscillations with lower mean vector length of spike phase compared to those in DMSO-injected mice (Fig. 3g–j, red). However, blue light stimulation of PV interneurons in AβO-injected PV-Cre mice (Fig. 3g–j, orange), but not SST interneurons in AβO-injected SST-Cre mice (Fig. 3g–j, green), restored the mean spike phase (Fig. 3i) and vector length of spike phase (Fig. 3j) similar to those in the DMSO-injected mice. Together, these results show that optogenetic activation of SST and PV interneurons selectively resynchronizes and increases the synchrony strength of the CA1 PC spikes relative to theta and gamma oscillations, respectively.

### Optogenetic activation of SST and PV interneurons resynchronizes SST and PV spike phases relative to theta and gamma oscillations, respectively, in AβO-injected mice in vivo

The generation of robust theta and gamma oscillations is thought to be helped by synchronized spiking of SST and PV interneurons at specific phases relative to ongoing oscillations (Amilhon et al. 2015; Gulyas et al. 2010; Huh et al. 2016; Mann et al. 2005; Mikulovic et al. 2018; Wang and Buzsaki 1996), which in turn synchronizes the CA1 PC activity (Mann and Paulsen 2007; Cobb et al. 1995; Zemankovics et al. 2013). Thus, we investigated the effect of AβO on spike phases of SST and PV interneurons relative to theta and gamma oscillations. First, we analyzed the probability distribution of SST and PV interneurons’ spike phases relative to the theta cycles in DMSO-injected mice and before/during blue light stimulation of SST and PV interneurons in AβO-injected mice in vivo. In DMSO-injected mice, SST interneuron preferentially spiked near the trough of the theta cycle (Fig. 4a–d, black), while PV interneuron preferentially spiked at the descending phase of the theta cycle (Fig. 4e–h, black), consistent with previous in vivo recordings in anesthetized states (Klausberger et al. 2003, 2004, 2005; Somogyi et al. 2014; Klausberger and Somogyi 2008). Interestingly, in AβO-injected mice, only SST interneuron spike phases were desynchronized relative to theta cycles showing advanced mean spike phase and reduced vector length of the spike phase (Fig. 4a–d, red), while those of PV interneurons were unaffected (Fig. 4e–h, red). Furthermore, blue light stimulation of SST interneurons in AβO-injected SST-Cre mice (Fig. 4a–d, green), but not PV interneurons in AβO-injected PV-Cre mice (Fig. 4e–h, orange), fully restored the mean spike phase (Fig. 4c, g) and vector length of the spike phase similar to those in the DMSO-injected mice (Fig. 4d, h).

We repeated the same spike phase analyses of SST and PV interneurons relative to gamma oscillations. In DMSO-injected mice, SST interneuron spiked near the trough of the gamma cycle (Fig. 4i–l, black), while PV interneuron preferentially spiked before the peak of the gamma cycle (Fig. 4m–p, black), consistent with previous in vivo recordings in anesthetized states (Somogyi et al. 2014; Tukker et al. 2007, 2013). In AβO-injected mice, the mean spike phase and the mean vector length of the spike phase of SST interneurons relative to gamma cycle were unaffected (Fig. 4i–l, red), while only those of PV interneurons were altered (Fig. 4m–p, red). Blue light stimulation of ChR2-expressing SST interneurons in AβO-injected SST-Cre mice had no significant effect on the mean spike phase and the mean vector length of the spike phase (Fig. 4i–l, green). However, blue light stimulation of ChR2-expressing PV interneurons in AβO-injected PV-Cre mice fully restored the mean spike phase and vector length of the spike phase similar to the effects observed in DMSO-injected mice (Fig. 4m–p, orange). These results revealed that AβO selectively desynchronized SST interneuron spikes relative to theta oscillations and PV interneuron spikes relative to gamma oscillations, which could be restored by optogenetic activation of SST and PV interneurons, respectively.

### Frequency-specific enhancement of sIPSC rates by optogenetic activation of SST and PV interneurons in AβO-injected mice ex vivo

Our results so far beg the question how sustained optogenetic activation of SST and PV interneurons can selectively resynchronize spike phases of CA1 PC (Fig. 3) and SST/PV interneurons (Fig. 4) relative to theta and gamma oscillations in a frequency-specific manner, thereby restoring impaired theta and gamma oscillations (Figs. 1, 2). Since precisely-timed inhibition provided by SST and PV interneurons is critical in theta and gamma oscillogenesis (Gulyas et al. 2010; Huh et al. 2016; Mann et al. 2005; Veit et al. 2017), we hypothesized that optogenetic activation of SST and PV interneurons may provide inhibition to CA1 PC in a frequency-selective manner. To test this, we obtained ex vivo whole-cell voltage-clamp recordings from CA1 PC in acute hippocampal slices of AβO-injected SST-Cre mice or PV-Cre mice and recorded sIPSCs during 3 s-long sustained blue light stimulation of ChR2-expressing SST interneurons (Fig. 5a, b) or ChR2-expressing PV interneurons (Fig. 5c, d). Analysis of the sIPSC rate as the total number of sIPSCs during the blue light stimulation revealed that blue light stimulation of SST interneurons generated sIPSCs in the theta frequency-range (Fig. 5e, green), while blue light stimulation of PV interneurons generated sIPSCs in the gamma frequency-range (Fig. 5e, orange). Analyses of band-pass filtered sIPSCs at theta frequency and the corresponding spectrograms (Fig. 5f, g) revealed that while the peak frequencies of sIPSCs were unaltered (Fig. 5h), the peak power of sIPSCs at theta frequency was increased only by optogenetic activation of ChR2-expressing SST interneurons but not by ChR2-expressing PV interneurons (Fig. 5i). In contrast, band-pass filtered sIPSCs at gamma frequency (Fig. 5j, k) revealed that while the peak frequency of sIPSCs was unchanged (Fig. 5l), the peak power of sIPSCs at gamma frequency was significantly increased only by optogenetic activation of ChR2-expressing PV interneurons, but not by ChR2-expressing SST interneurons (Fig. 5m). Similar enhancements of the peak power of sIPSCs at theta and gamma frequencies by optogenetic activation of ChR2-expressing SST and PV interneurons, respectively, were observed in ex vivo brain slices from DMSO-injected SST-Cre/PV-Cre mice (Online resource Fig. 5). These results indicate that optogenetic activation of SST and PV interneurons increases sIPSC rates onto CA1 PC in a frequency-specific manner, contributing to the enhancement of the peak power of theta and gamma oscillations, respectively, in both, models of AD and the healthy brain.

### AβO induces frequency-specific presynaptic dysfunctions of SST and PV interneurons’ input to CA1 PC

Then, what are the synaptic mechanisms underlying frequency-selective impairments of theta and gamma oscillations induced by AβO? Desynchronized SST and PV interneurons’ firing relative to oscillations in AβO-injected mice (Fig. 4) suggests that AβO may have decreased the strength of SST/PV interneuron-evoked inhibitory input into CA1 PC. Moreover, enhancement of sIPSC powers at theta and gamma frequencies by optogenetic activation of SST and PV interneurons ex vivo, respectively (Fig. 5), indicates that the optogenetic activation might restore the amplitude of sIPSCs impaired by AβO. Thus, we hypothesized that AβO disrupts SST and PV interneuron-evoked synaptic inputs to CA1 PC leading to a desynchronization of CA1 PC spikes. To test this hypothesis, we conducted ex vivo whole-cell voltage-clamp recordings from CA1 PC and recorded IPSCs evoked by a single or repetitive brief pulses of blue light stimulation of ChR2-expressing SST (SST-evoked IPSCs_,_ Fig. 6a, b) and PV interneurons (PV-evoked IPSCs_,_ Fig. 6c, d) in hippocampal slices from DMSO-injected and AβO-injected SST-Cre/PV-Cre mice. We first analyzed the *S*–*R* curve of SST-evoked IPSCs (Fig. 6b) and PV-evoked IPSCs (Fig. 6d) in response to a single pulse of different blue light intensities [5, 10, 25, 50, 75, and 100% of maximal light power (15 mW)]. In ex vivo hippocampal slices from AβO-injected mice, the amplitudes of both SST-evoked IPSCs and PV-evoked IPSCs were significantly increased at each given light intensity, compared to those in DMSO-injected mice (Fig. 6b, d, right), which indicates that AβO altered both SST-to-PC and PV-to-PC synapses to enhance the initial GABA release probability in AβO-injected mice. Interestingly, the *S*–*R* curves of both SST- and PV-evoked IPSCs in the AβO-injected mice reached significantly higher plateaus than in the DMSO-injected mice at high light intensities (Fig. 6b, d, right), which argues against the possibility that the difference in IPSC sizes is due to a change in optogenetic stimulation-induced increase in interneuron excitability, as we would then expect the *S*–*R* curves to eventually reach the same plateau. Since SST and PV interneurons’ inhibitory inputs to CA1 PC were tuned at theta and gamma frequencies, respectively (Fig. 5), we further investigated whether AβO-induced synaptic dysfunctions at SST-to-PC and PV-to-PC synapses had frequency-dependence. To test this, we delivered ten pulses of blue light at 5 Hz and 40 Hz in ex vivo slices from DMSO-injected and AβO-injected mice to record SST-evoked IPSCs (Fig. 6e, i, black traces) and PV-evoked IPSCs (Fig. 6g, k, black traces) and analyzed PPR and short-term plasticity (Fig. 6f, h, j, l). In doing so, we used half-maximal blue light stimulation intensity based on the *S*–*R* curve (Fig. 6b, d) to ensure that the light stimulation intensity does not affect the neurotransmitter release probability, a method widely used in investigating synaptic properties (Ciani et al. 2015; He et al. 2019; Rice et al. 2019; Abramov et al. 2009; Phillips et al. 2008). When we analyzed the first two-consecutive IPSCs evoked by SST or PV interneurons, we found that paired-pulse depression of SST-evoked IPSCs, as observed in slices from DMSO-injected mice, was further depressed only for 5 Hz stimulation (Fig. 6f, j, left), while paired-pulse depression of PV-evoked IPSCs was further depressed only for 40 Hz stimulation (Fig. 6h, l, left). Similarly, when all ten pulses were analyzed for short-term plasticity, SST-evoked IPSCs and PV-evoked IPSCs at both 5 and 40 Hz showed robust short-term depression (Fig. 6f, h, j, l, right, black) of IPSCs in slices from DMSO-injected slices, while in hippocampal slices from AβO-injected mice, short-term depression was further depressed only at 5 Hz for SST-evoked IPSCs (Fig. 6f, j, right, red) and only at 40 Hz for PV-evoked IPSCs (Fig. 6h, l, right, red). These results indicate that AβO causes presynaptic dysfunctions at SST-to-PC and PV-to-PC synapses by increasing the initial GABA release probability to depress SST-evoked and PV-evoked IPSCs selectively at theta and gamma frequencies, respectively. Finally, we asked whether AβO-induced dysfunctions of SST and PV interneurons’ inhibitory inputs to CA1 PC in AβO-injected mice could be restored by the sustained optogenetic activation of SST and PV interneurons. For this, we analyzed the mean amplitude of sIPSCs recorded in ex vivo slices from DMSO-injected mice (Fig. 6m, top), AβO-injected mice (Fig. 6m, bottom), and AβO-injected mice during sustained blue light stimulation of ChR2-expressing SST interneuron (Fig. 6n, top) and ChR2-expressing PV interneuron (Fig. 6n, bottom). Compared to the sIPSCs recorded in ex vivo slices from DMSO-injected mice, the mean amplitudes of sIPCSs were significantly reduced in ex vivo slices from AβO-injected mice (Fig. 6o, p). Such AβO-induced reduction of sIPSC amplitude was fully restored by optical stimulation of SST and PV interneurons (Fig. 6o, p). Taken together, these results demonstrate for the first time that AβO causes presynaptic dysfunction at SST-to-PC and PV-to-PC synapses by increasing the initial synaptic release probabilities, which leads to a frequency-specific and cell type-specific depression in SST-evoked IPSCs and PV-evoked IPSCs. Such AβO-induced depression of inhibitory input to CA1 PC may underlie the desynchronization of CA1 PC spikes, leading to a reduction in theta and gamma oscillation powers in vivo in AβO-injected mice.

## Discussion

In this study, we injected AβO into the hippocampus to create AβO pathology in vivo, a method adopted in many studies investigating the impact of AβO on physiological and cognitive functions (Villette et al. 2010; Meyer-Luehmann et al. 2006; Stephan et al. 2001, 2003; Walsh et al. 2002). The major advantage of using the AβO-injection mouse model of AD was that we were able to perform optogenetic activation of SST and PV interneurons in *cre*-transgenic animals. Using this AD mouse model, we showed, for the first time, that sustained optogenetic activation of SST and PV interneurons could selectively restore the power of hippocampal theta and gamma oscillations impaired by AβO pathology in vivo, respectively, (Figs. 1, 2) without affecting other frequencies such as delta and beta oscillations (Online resource Fig. 2), and resynchronize the CA1 PC spike phases relative to both theta and gamma oscillations to the level observed in the control mice (Fig. 3). Further analyses revealed that SST and PV interneurons’ spike phases desynchronized specifically relative to theta and gamma oscillation cycles, respectively (Fig. 4), which were also resynchronized by the optogenetic manipulations. Ex vivo whole-cell patch-clamp recordings of sIPSCs from CA1 PC in hippocampal slices prepared from AβO-injected mice revealed that optogenetic activation of SST and PV interneurons selectively enhanced sIPSC rates at theta and gamma frequencies, respectively (Fig. 5), providing a mechanisms for the observed restoration of theta and gamma oscillations in vivo. Moreover, analysis of the *S*–*R* curve, PPR, and short-term plasticity of SST-evoked and PV-evoked IPSCs showed that AβO induced presynaptic dysfunctions at SST-to-PC and PV-to-PC synapses by depressing SST and PV interneurons’ input to CA1 PC in a frequency-specific manner, accounting for the reduction in the power of theta and gamma oscillations, respectively. Taken together, our results provide mechanistic insight into the inhibitory neural circuit dysfunctions underlying AβO-induced theta and gamma oscillation impairments in vivo and identify SST and PV interneurons as targets for restoring hippocampal network oscillation impairments in early AD.

Hippocampal gamma and theta oscillations, which are important in memory function, have been reported to be impaired in mouse models of AD (Wang et al. 2002; Scott et al. 2012; Villette et al. 2010; Iaccarino et al. 2016; Driver et al. 2007; Palop and Mucke 2016; Goutagny et al. 2013; Ittner et al. 2014; Mondragon-Rodriguez et al. 2018). Thus, many previous studies investigated ways in which hippocampal oscillations could be restored. PV interneurons have long been implicated in gamma oscillations (Gulyas et al. 2010; Mann et al. 2005), and consequently their activity has been manipulated to restore gamma oscillations in mouse models of AD through methods such as optogenetic activation of PV interneurons (Iaccarino et al. 2016), implantation of PV-like fast-spiking interneurons (Martinez-Losa et al. 2018; Tong et al. 2014) and even ablation of certain genes in PV interneurons (Verret et al. 2012; Zhang et al. 2017), which are in line with our results. However, no study has yet been able to suggest methods to restore theta oscillation impairments observed in mouse models of AD. As for SST interneurons, their roles on hippocampal oscillogenesis appear more varied (Amilhon et al. 2015; Huh et al. 2016; Mikulovic et al. 2018). Some studies report that the activity of SST interneurons is synchronized with gamma oscillations in vivo (Tukker et al. 2007) and optogenetic activation of SST interneurons can modulate gamma oscillations in vivo and ex vivo (Hakim et al. 2018; Veit et al. 2017). Recently, optogenetic activation of O-LM cells, a subset of SST interneurons, were shown to induce theta frequency oscillations in vivo in awake behaving animals (Mikulovic et al. 2018). Consistent with these results, our in vivo (Online resource Fig. 3) and ex vivo experiments (Online resource Fig. 5) demonstrate that optogenetic activation of SST interneurons enhances theta oscillations in healthy brains. To our knowledge, this is the first demonstration that optogenetic activation of SST interneurons selectively restores theta oscillations and resynchronizes CA1 PC spikes relative to theta oscillations in a mouse model of AD in vivo by selectively restoring the dysfunctional SST interneuronal input to CA1 PC.

Through spike phase analyses of CA1 PC relative to theta and gamma frequency oscillations, we were able to identify that their spike phases were desynchronized in AβO-injected mice (Fig. 3), which is consistent with other studies (Kurudenkandy et al. 2014; Mably et al. 2017). However, we also found that, in AβO-injected mice, SST and PV interneurons’ synchrony strengths were selectively reduced relative to theta and gamma oscillations, respectively (Fig. 4). These results led us to show that AβO causes interneuron subtype-specific and frequency-specific impairments of inhibitory input to CA1 PC, as further confirmed through experiments ex vivo (Figs. 5, 6).

We found that sustained optogenetic activation of SST and PV interneurons in AβO-injected mice increase sIPSC rates selectively at theta and gamma frequencies, respectively (Fig. 5). These results indicate that optogenetic activation of these interneurons restored neuronal output in a frequency-specific manner, which contributed to the restoration of theta and gamma oscillations in vivo in Figs. 1 and 2. In fact, such frequency-specific restoration of theta and gamma oscillations could be in part explained by additional excitation provided by optogenetic activation of SST/PV interneurons allowing for the activation of SST/PV interneuronal networks to a critical level for oscillations, which would otherwise show reduced activation due to the AβO-induced decrease in the excitation level in SST and PV interneurons (Hanson 2017; Schmid et al. 2016; Park et al. 2020). Indeed, optogenetic activation of SST and PV interneurons restored the sIPSC amplitudes to the level observed in DMSO-injected mice (Fig. 6) by increasing excitation to SST and PV interneurons. This led to increased spontaneous firing of SST or PV interneurons, similar to what has been reported in other studies (Song et al. 2013; Yi et al. 2014).

To better dissect the synaptic mechanisms underlying AβO-induced impairments of theta and gamma oscillations, we analyzed the *S*–*R* curve, PPR, and short-term plasticity of SST/PV interneuron-evoked IPSCs in ex vivo slices and were able to show that AβO enhances both paired-pulse depression and short-term depression of SST/PV interneuron-evoked IPSCs (Fig. 6). These results suggest that AβO increases the initial synaptic release probability of SST and PV interneurons resulting in a depression of their synaptic weights to CA1 PC. This effect selectively occurs for SST interneurons at theta and PV interneurons at gamma frequencies, which may explain AβO-induced impairment of theta and gamma oscillations as well as desynchronization of PC, SST and PV interneurons’ spike phases. However, we cannot rule out the possibility that these frequency-selective presynaptic dysfunctions of SST/PV interneurons inputs to CA1 PC might result from changes in the level of activation of SST/PV interneurons or in the resonant properties of the network by the repeated light pulses. In addition, since activation of SST interneurons has been shown to recruit PV interneurons (Cottam et al. 2013; Xu et al. 2013) or vice versa (Karnani et al. 2016; Walker et al. 2016), it is possible that optogenetic activation of SST/PV interneurons could have restored the amplitudes of sIPSCs by altering the number of co-active SST/PV interneurons. Thus, future experiments will require dual recordings of interneuron and PC pairs to ascertain the presynaptic dysfunctions we describe here.

Although our study dissects the contributions of SST and PV interneurons to theta and gamma oscillations in an AD-model and healthy brains, one thing to note in interpreting our data is that all in vivo recordings were performed under anesthesia. While there are reports that the power of theta oscillations was reduced in transgenic mouse models of AD such as 3xTg ex vivo (Mondragon-Rodriguez et al. 2018), CRND8 ex vivo (Goutagny et al. 2013), APP/PS1 in vivo (Wang et al. 2002; Scott et al. 2012), APP23 in vivo (Ittner et al. 2014), one study reported that theta oscillations during spatial navigation in awake behaving mice was unaffected in 5XFAD mice in vivo (Zhang et al. 2016). These differences may arise depending on the behavioral states and differences in the transgenic mouse lines of AD. Therefore, it will be important to investigate how theta oscillations are affected in the awake AβO-injected mice and the effectiveness of optogenetic activation of SST and PV interneurons thereon. Moreover, hypersynchrony and epileptic activities observed in transgenic AD mice models (Palop et al. 2007) was absent in the AβO-injected mice in our and other studies (Kalweit et al. 2015). Hence, long-term effects of AβO may also cause different impairments on hippocampal neural circuits, which will require further investigation.

Overall, by combining optogenetic manipulations of SST and PV interneurons in a AβO-injection mouse model of AD in vivo and ex vivo, we dissected the neural circuit dysfunction underlying theta and gamma oscillation impairments caused by AβO pathology and show that hippocampal theta and gamma oscillations can be selectively restored by optogenetic activation of SST and PV interneurons, respectively. These results provide experimental evidence for how SST and PV interneurons contribute to theta and gamma oscillogenesis in the hippocampus and suggest that they could serve as potential therapeutic targets for restoring hippocampal oscillations in early stages of AD.

## Electronic supplementary material

Below is the link to the electronic supplementary material.

Supplementary file1 (DOCX 1681 kb)
