# Supplementary material for: Dissociation of somatostatin and parvalbumin interneurons circuit dysfunctions underlying hippocampal theta and gamma oscillations impaired by amyloid β oligomers in vivo
Source: Brain Struct Funct. 2020 Feb 27;225(3):935–54. doi: 10.1007/s00429-020-02044-3 (PMC7166204; doi:10.1007/s00429-020-02044-3)
Supplement: Supplementary file 1 — Supplementary file1 (DOCX 1681 kb) [file 429_2020_2044_MOESM1_ESM.docx]

**Brain Structure and Function**

**Supplementary material**

**Dissociation of somatostatin and parvalbumin interneurons circuit dysfunctions underlying hippocampal theta and gamma oscillations impaired by amyloid β oligomers *in vivo***

**Hyowon Chung^1,^** ^†^**, Kyerl Park^1,^** ^†^**, Hyun Jae Jang^1^, Michael M Kohl^2^**

**and Jeehyun Kwag^1*^**

**Affiliations:**

^1^Department of Brain and Cognitive Engineering, Korea University, Seoul, Korea

^2^Department of Physiology, Anatomy and Genetics, University of Oxford, Oxford, UK

^†^Hyowon Chung and Kyerl Park contributed equally to this work.

*To whom correspondence may be addressed.

Email: jkwag@korea.ac.kr.

Dr. Jeehyun Kwag

Department of Brain and Cognitive Engineering, Korea University,

145 Anam-ro, Seungbuk-gu, Seoul, Korea

Email: jkwag@korea.ac.kr

Office: +82-2-3290-5924


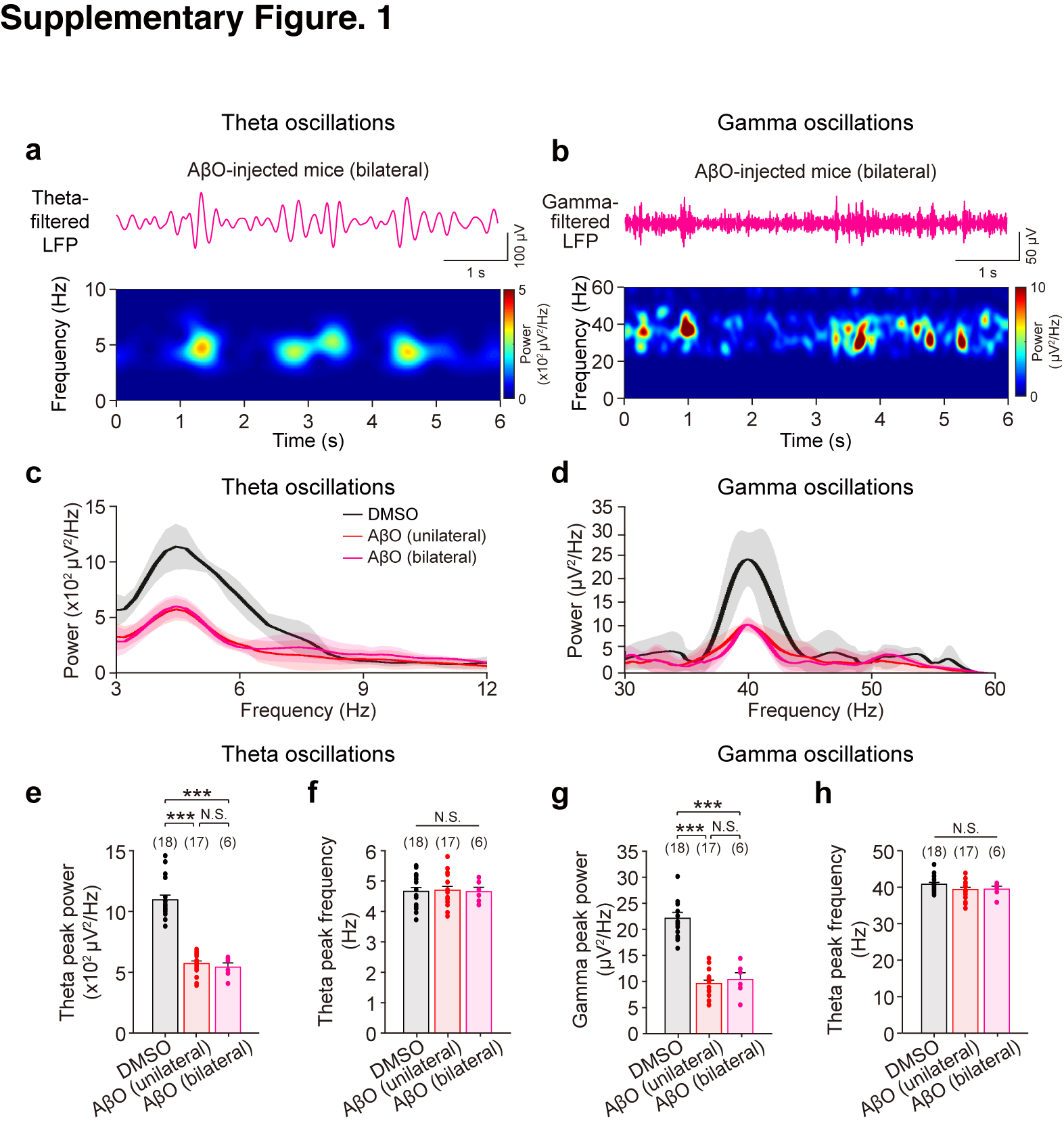


**Supplementary Figure 1** Effect of unilateral and bilateral hippocampal injection of AβO on hippocampal theta and gamma oscillation impairments *in vivo*. **a, b** Representative traces of band-pass filtered LFPs (top) at theta (**a**) and gamma frequencies (**b**) and the corresponding power spectrograms (bottom) recorded in mice injected with AβO in both hippocampi bilaterally. **c, d** Power spectral density (PSD) of unfiltered LFPs (shade indicates SEM) in theta (3-12 Hz, **c**) and gamma frequency-range (30-60 Hz, **d**). **e, f** Mean peak power (**e**) and mean peak frequency (**f**) of theta oscillations analyzed from the PSD of unfiltered LFPs recorded in mice injected with DMSO in the left hippocampus unilaterally (n = 18, black, from Fig. 1, 2), in mice injected with AβO in the left hippocampus unilaterally (n = 17, red, from Fig. 1, 2), and in mice injected with AβO in both hippomcampi bilaterally (n = 6, magenta). **g, h** Same as (**e, f**) but for gamma oscillations in each condition. n indicates the number of animals from which LFPs were recorded. Data are mean ± SEM with individual data values (dots). One-way ANOVA followed by Tukey’s *post hoc* test (**e**, **f**, **g**, **h**. *** *p* < 0.001, N.S. *p* > 0.05).

**
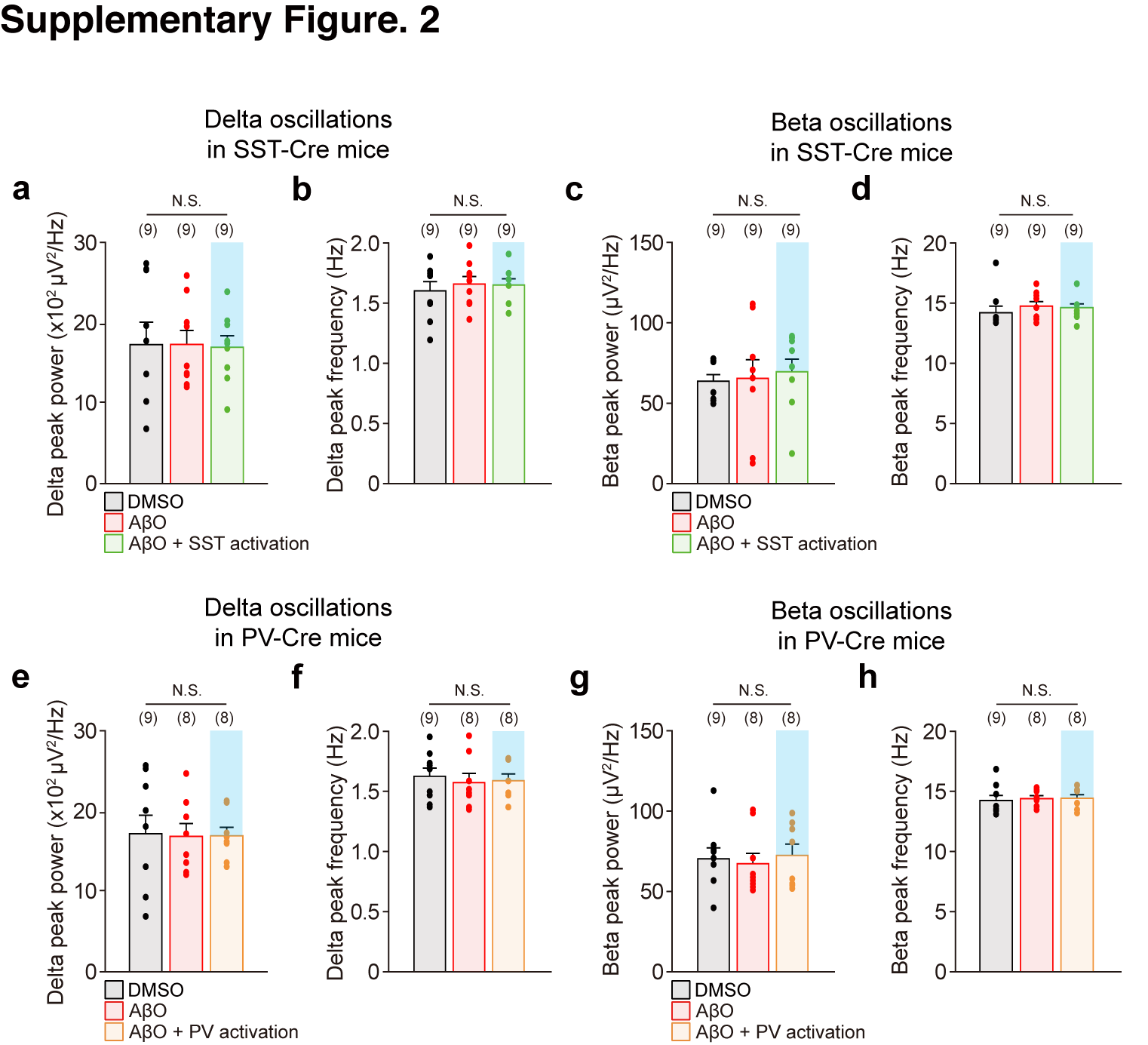
**

**Supplementary Figure 2** Effect of optogenetic activation of SST+ and PV+ interneurons on delta and beta oscillations in AβO-injected mice *in vivo*. **a, b** Mean peak power (**a**) and mean peak frequency (**b**) of delta oscillations analyzed from power spectral density (PSD) of unfiltered LFPs (shown in Fig. 1n) recorded in DMSO-injected SST-Cre mice (n = 9, black), in AβO-injected SST-Cre mice (n = 9, red), and with blue light (473 nm) stimulation of ChR2-expressing SST+ interneurons in AβO-injected SST-Cre mice (n = 9, green). **c, d** Same as (**a, b**) but for beta oscillations. **e, f** Mean peak power (**e**) and mean peak frequency (**f**) of delta oscillations analyzed from PSD of unfiltered LFPs (shown in Fig. 2g) recorded in DMSO-injected PV-Cre mice (n = 9, black), in AβO-injected PV-Cre mice (n = 8, red), and with blue light stimulation of ChR2-expressing PV+ interneurons in AβO-injected PV-Cre mice (n = 8, orange). **g, h** Same as (**e, f**) but for beta oscillations. n indicates the number of animals from which LFPs were recorded. Data are mean ± SEM with individual data values (dots). One-way ANOVA followed by Tukey’s *post hoc* test (**a**-**h**, N.S. *p* > 0.05).


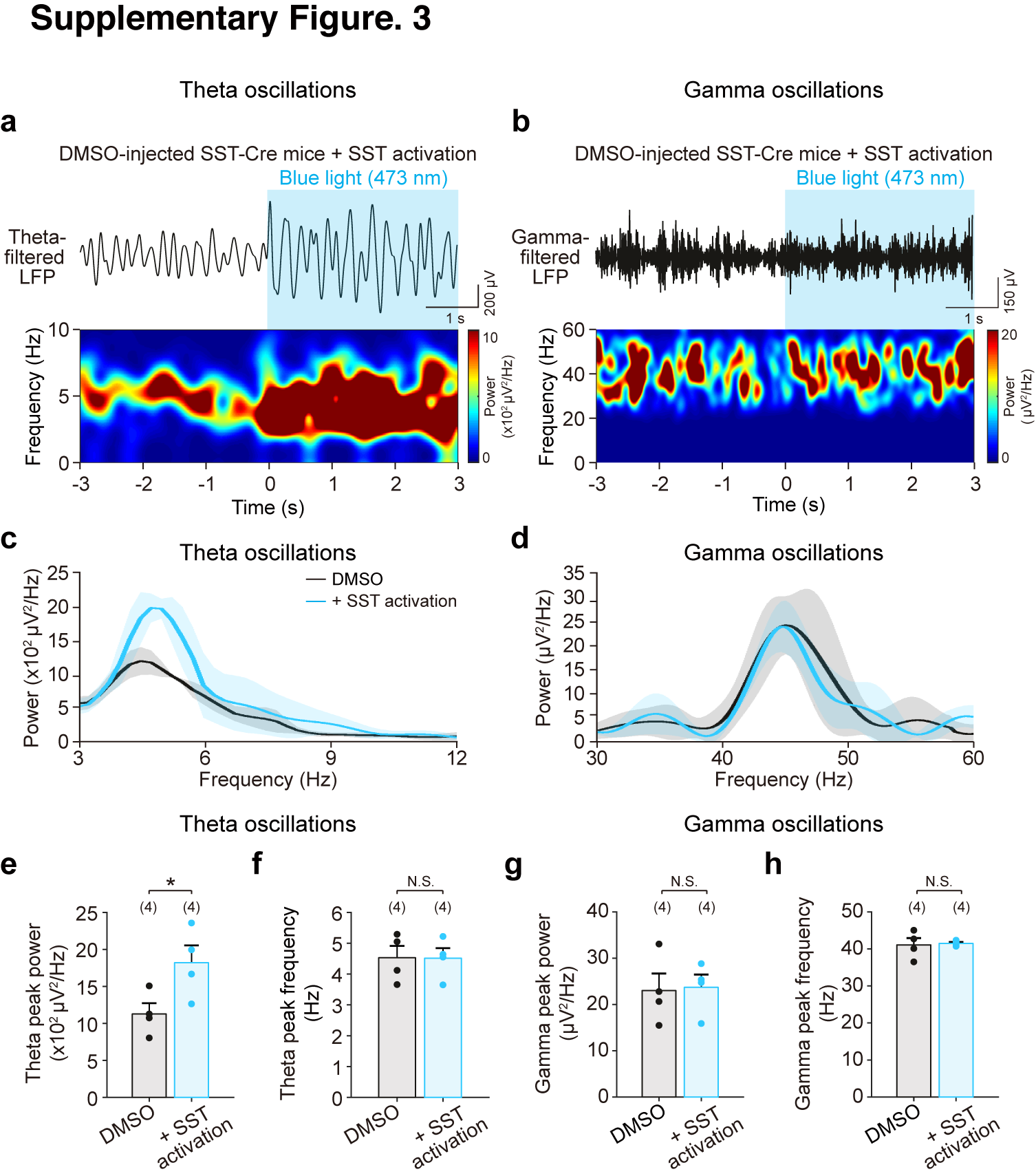


**Supplementary Figure 3** *In vivo* optogenetic activation of SST+ interneurons selectively increases the power of theta oscillations in DMSO-injected mice. **a** Representative trace of band-pass filtered LFPs at theta frequency (top) and the corresponding power spectrogram (bottom) before and during blue light (473 nm) stimulation (blue shade) of ChR2-expressing SST+ interneurons in DMSO-injected SST-Cre mice. **b** Same as **a** but for band-pass filtered LFPs at gamma frequency. **c, d** Power spectral density (PSD) of unfiltered LFPs (shade indicates SEM) in theta (**c**) and gamma frequency-range (**d**) recorded in DMSO-injected SST-Cre mice (n = 4, black) and during blue light stimulation of ChR2-expressing SST+ interneurons (n = 4, blue). **e, f** Mean peak power (**e**) and mean peak frequency (**f**) of theta oscillations analyzed from PSD in **c**. **g, h** Same as (**e, f**) but for gamma oscillations analyzed from PSD in **d**. n indicates the number of animals from which LFPs were recorded. Data are mean ± SEM with individual data values (dots). Paired Student’s *t*-test (**e**-**h**, * *p* < 0.05, N.S. *p* > 0.05).


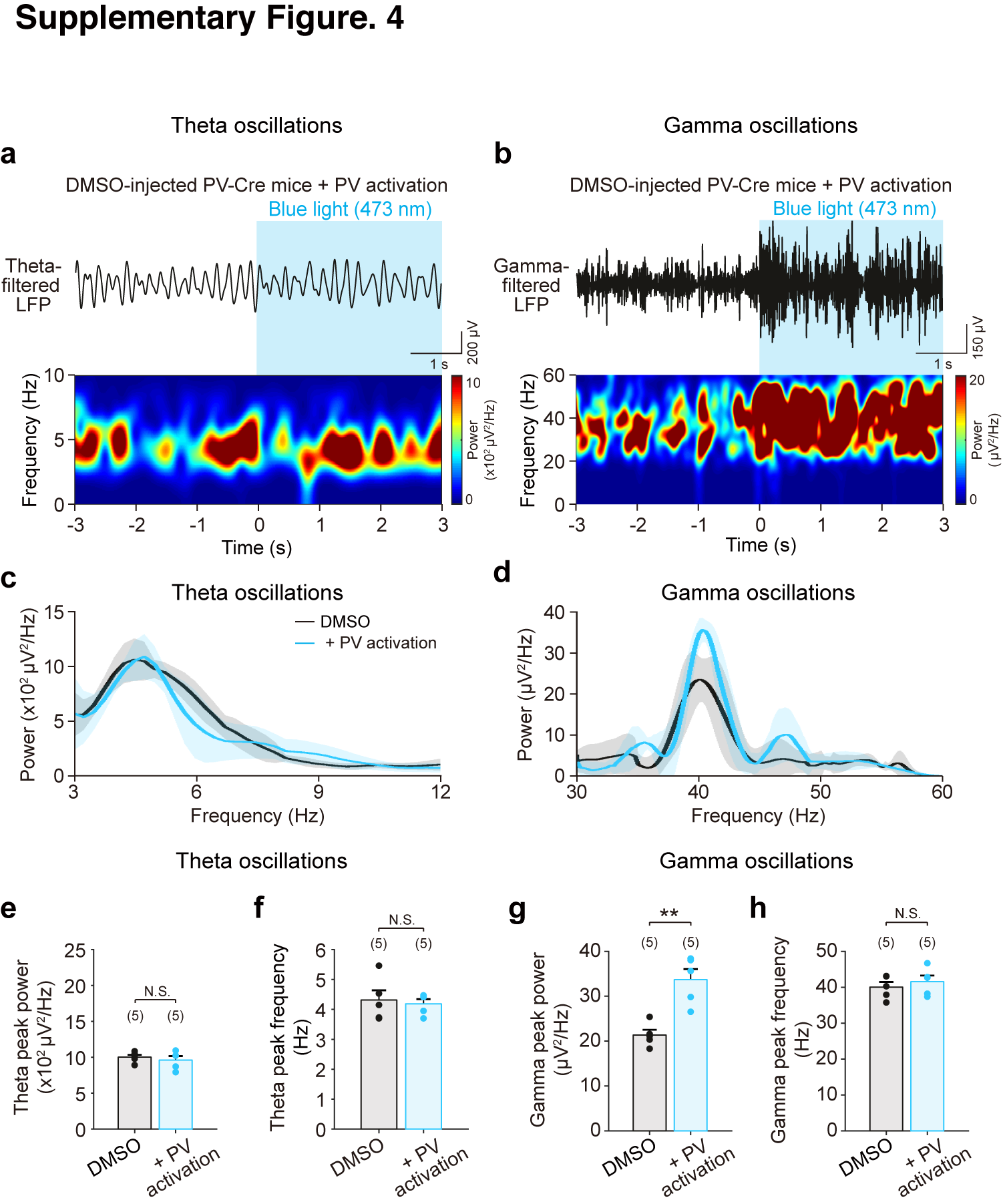


**Supplementary Figure 4** *In vivo* optogenetic activation of PV+ interneurons selectively increases the power of gamma oscillations in DMSO-injected mice. **a** Representative trace of band-pass filtered LFPs at theta frequency (top) and the corresponding power spectrogram (bottom) before and during blue light (473 nm) stimulation (blue shade) of ChR2-expressing PV+ interneurons in DMSO-injected PV-Cre mice. **b** Same as **a** but for band-pass filtered LFPs at gamma frequency. **c, d** Power spectral density (PSD) of unfiltered LFPs (shade indicates SEM) in theta (**c**) and gamma frequency-range (**d**) recorded in DMSO-injected PV-Cre mice (n = 5, black) and during blue light stimulation of ChR2-expressing PV+ interneurons (n = 5, blue). **e, f** Mean peak power (**e**) and mean peak frequency (**f**) of theta oscillations analyzed from PSD in **c**. **g, h** Same as (**e, f**) but for gamma oscillations analyzed from PSD in **d**. n indicates the number of animals from which LFPs were recorded. Data are mean ± SEM with individual data values (dots). Paired Student’s *t*-test (**e**-**h,** ** *p* < 0.01, N.S. *p* > 0.05).


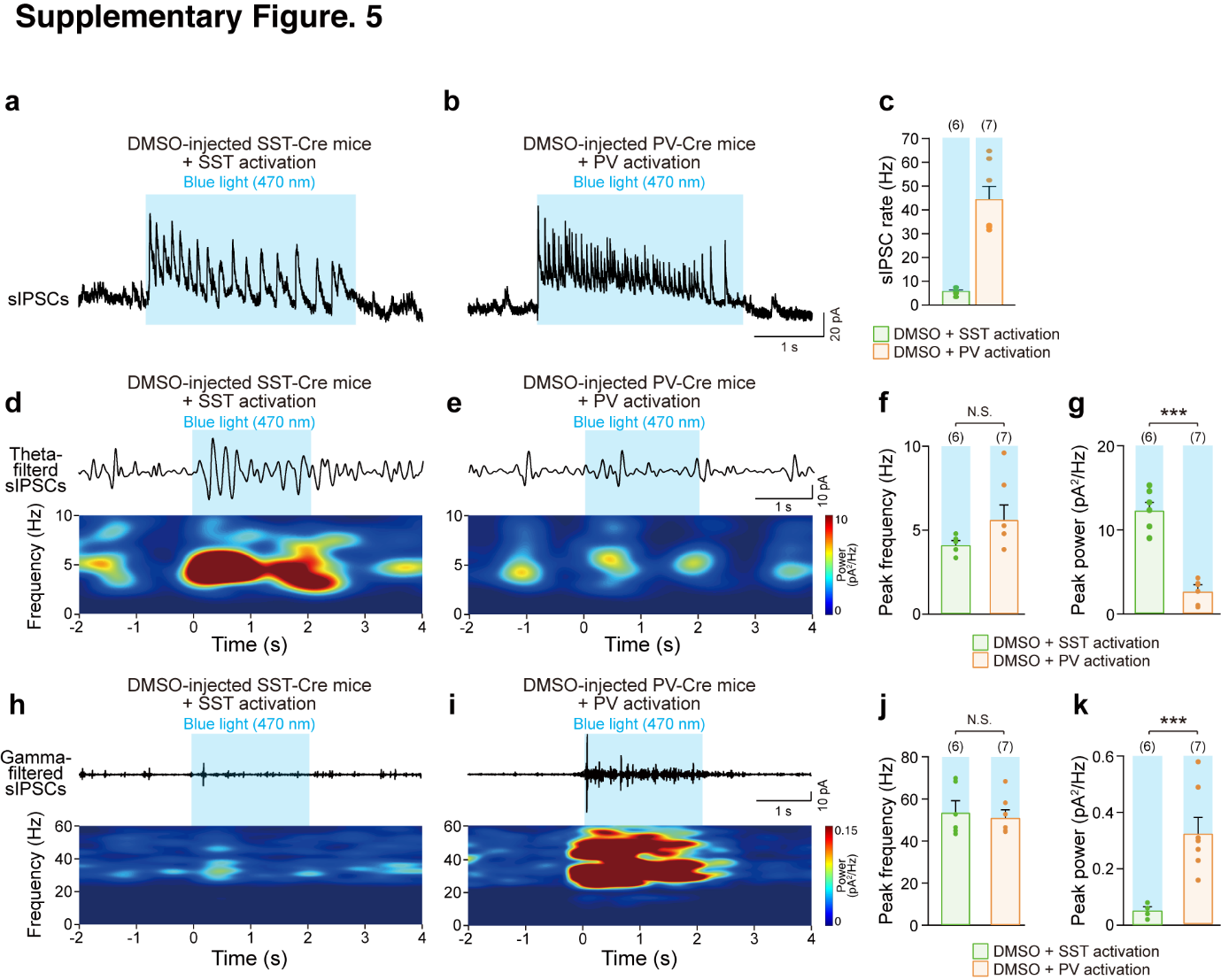


**Supplementary Figure 5** *Ex vivo* optogenetic activation of SST+ and PV+ interneurons in DMSO-injected mice selectively enhances sIPSCs at theta and gamma frequencies, respectively. **a, b** Representative traces of *ex vivo* whole-cell voltage-clamp recordings of sIPSCs from CA1 PC during sustained blue light (470 nm) stimulation (blue shade) of ChR2-expressing SST+ interneurons in *ex vivo* hippocampal slices from DMSO-injected SST-Cre mice (**a**) and ChR2-expressing PV+ interneurons in slices cut from DMSO-injected PV-Cre mice (**b**). **c** Mean rates of sIPSCs in each condition (DMSO + SST activation: n = 6, DMSO + PV activation: n = 7). **d, e** Representative traces of band-pass filtered sIPSCs at theta frequency (top) and the corresponding power spectrograms (bottom) during sustained blue light stimulation of ChR2-expressing SST+ interneurons (**d**) and ChR2-expressing PV+ interneurons (**e**) in *ex* vivo hippocampal slices from DMSO-injected SST-Cre mice and PV-Cre mice, respectively. **f, g** Mean peak frequency (**f**) and mean peak power (**g**) of band-pass filtered sIPSCs at theta frequency during blue light stimulation of ChR2-expressing SST+ interneurons in DMSO-injected SST-Cre mice (n = 6, green) and ChR2-expressing PV+ interneurons in DMSO-injected PV-Cre mice (n = 7, orange). **h-k** Same as (**d-g**) but for band-pass filtered sIPSCs at gamma frequency. n indicates the number of cells from which sIPSCs were recorded. Data are mean ± SEM with individual data values (dots). Paired Student’s *t*-test (**f**, **g**, **j**, **k**, *** *p* < 0.001, N.S. *p* > 0.05).
